# Chronic kidney disease stages and changes in ambulatory blood pressure monitoring: letter to the editor

**DOI:** 10.1590/2175-8239-JBN-2024-0128en

**Published:** 2024-10-11

**Authors:** Hineptch Daungsupawong, Viroj Wiwanitkit

**Affiliations:** 1 Private Academic and Editorial Consultant, Phonhong, Laos.; 2 University Centre for Research & Development Department of Pharmaceutical Sciences, Chandigarh University Gharuan, Mohali, Punjab, India.

Dear Editor,

We would like to share ideas on the publication entitled “Association between chronic kidney disease stages and changes in ambulatory blood pressure monitoring parameters^1^.” The purpose of the study was to assess the relationships between changes in ambulatory blood pressure monitoring (ABPM) parameters and various stages of chronic kidney disease (CKD). A total of 851 participants from outpatient clinics of a university hospital who had ABPM exams were included in the cross-sectional study. The findings demonstrated that only stage 5 CKD was associated with pulse pressure, while systolic blood pressure (SBP) was associated with both stages 3b and 5. Stages 3a, 4, and 5 were gradually associated to the SBP coefficient of variation, suggesting a connection between CKD progression and SBP variability.

The cross-sectional nature of the data in this study is a weakness that could hinder the demonstration of causal relationships between ABPM parameters and CKD stages. Furthermore, the study only included patients from one university hospital, which means that the participants may not be entirely typical of the CKD community as a whole. It would be crucial to take into account additional potential confounding variables, such as dietary habits, level of physical activity, and comorbidities, which were not accounted for in the study.

Potential questions for the author are how changes in medication consumption over the study period were taken into account in the analysis and what precise criteria were utilized to place patients into different stages of CKD. Longitudinal studies may be used in this field in the future to gain a deeper understanding of the temporal link between changes in ABPM parameters and the progression of CKD. Furthermore, examining the effects of various hypertension treatment approaches in CKD patients could improve care for this population.
