# Supplementary material for: Chronic kidney disease stages and changes in ambulatory blood pressure monitoring: letter to the editor
Source: J Bras Nefrol. 2024 Oct 11;46(4):e20240128. doi: 10.1590/2175-8239-JBN-2024-0128en (PMC11469576; doi:10.1590/2175-8239-JBN-2024-0128en)
Supplement: Translation

# Translation: Estágios da doença renal crônica e alterações na monitorização ambulatorial da pressão arterial: carta ao editor

**DOI:** 10.1590/2175-8239-JBN-2024-0128pt

**Authors:** Hineptch Daungsupawong, Viroj Wiwanitkit

**Affiliations:** 1 Private Academic and Editorial Consultant, Phonhong, Laos.; 2 University Centre for Research & Development Department of Pharmaceutical Sciences, Chandigarh University Gharuan, Mohali, Punjab, India.

Prezado editor,

Gostaríamos de compartilhar nossas considerações sobre a publicação intitulada “Associação entre estágios da doença renal crônica e alterações dos parâmetros da monitorização ambulatorial da pressão arterial”^1^. O objetivo do estudo foi avaliar as relações entre as alterações dos parâmetros de monitorização ambulatorial da pressão arterial (MAPA) e os diferentes estágios da doença renal crônica (DRC). Foram incluídos no estudo transversal 851 participantes de clínicas ambulatoriais de um hospital universitário que realizaram exames de MAPA. Os achados demonstraram que somente o estágio 5 da DRC foi associado à pressão de pulso, enquanto a pressão arterial sistólica (PAS) foi associada aos estágios 3b e 5. Os estágios 3a, 4 e 5 foram gradualmente associados ao coeficiente de variação da PAS, sugerindo uma conexão entre a progressão da DRC e a variabilidade da PAS.

A natureza transversal dos dados deste estudo é uma limitação que pode dificultar a demonstração de relações causais entre os parâmetros da MAPA e os estágios da DRC. Além disso, o estudo incluiu somente pacientes de um hospital universitário, o que significa que os participantes podem não ser totalmente representativos da comunidade de pacientes com DRC como um todo. Seria crucial considerar outras variáveis de confusão em potencial, como hábitos alimentares, nível de atividade física e comorbidades, que não foram contempladas no estudo.

Perguntas potenciais para o autor incluem como alterações no consumo de medicamentos durante o período do estudo foram consideradas na análise e quais critérios precisos foram utilizados para classificar os pacientes nos diferentes estágios da DRC. Estudos longitudinais podem ser utilizados futuramente nessa área para obter uma compreensão mais abrangente da relação temporal entre as alterações dos parâmetros da MAPA e a progressão da DRC. Além disso, examinar os efeitos das diversas abordagens de tratamento da hipertensão em pacientes com DRC pode melhorar o atendimento a essa população.
